# SPARX, a MIMO Array for Ground-Based Radar Interferometry

**DOI:** 10.3390/s19020252

**Published:** 2019-01-10

**Authors:** Alberto Michelini, Francesco Coppi, Alberto Bicci, Giovanni Alli

**Affiliations:** 1 IDS GeoRadar, 56121 Pisa, Italy; francesco.coppi@idsgeoradar.com (F.C.); alberto.bicci@idsgeoradar.com (A.B.); 2 IDS Ingegneria Dei Sistemi S.p.A., 56121 Pisa, Italy; g.alli@idscorporation.com

**Keywords:** GB-SAR, MIMO radar, radar imaging

## Abstract

Ground-Based SAR Interferometry (GB-InSAR) is nowadays a proven technique widely used for slope monitoring in open pit mines and landslide control. Traditional GB-InSAR techniques involve transmitting and receiving antennas moving on a scanner to achieve the desired synthetic aperture. Mechanical movement limits the acquisition speed of the SAR image. There is a need for faster acquisition time as it plays an important role in correcting rapidly varying atmospheric effects. Also, a fast imaging radar can extend the applications to the measurement of vibrations of large structures. Furthermore, the mechanical assembly put constraints on the transportability and weight of the system. To overcome these limitations an electronically switched array would be preferable, which however faces enormous technological and cost difficulties associated to the large number of array elements needed. Imaging Multiple-Input Multiple Output (MIMO) radars can be used as a significant alternative to usual mechanical SAR and full array systems. This paper describes the ground-based X-band MIMO radar SPARX recently developed by IDS GeoRadar in order to overcome the limits of IDS GeoRadar’s well-established ground based interferometric SAR systems. The SPARX array consists of 16 transmit and 16 receive antennas, organized in independent sub-modules and geometrically arranged in order to synthesize an equally spaced virtual array of 256 elements.

## 1. Introduction

Nowadays, thanks to its distinguishing features [1,2] GB-InSAR technology has become a consolidated technique for measure ground displacements in many geophysical applications [2,3,4] and has proved to be particularly suitable in environments where continuous and real-time monitoring is required [5,6]. Despite the high number of technological advances seen in the last decade, some typical limitations are still present in the standard GB-InSAR systems, and therefore many improvements can be performed with respect to the current technique.

Some of the persisting GB-InSAR limitations are related to its mechanical scanning. In fact, traditional GB-InSAR techniques require a radar sensor equipped with transmitting and receiving antennas, moved by a mechanical scanner to achieve the desired synthetic aperture [7]. This approach, even if it has proved to be simple and effective, can have a considerable impact on important operational aspects such as scanning times, maintenance and installation, which will be discussed briefly below.

The data acquisition time is one of the most important parameters in the evaluation of a remote sensing monitoring system; since the first GB-InSAR introduction, the scanning times have been significantly reduced, and currently the fastest systems can scan the entire 360° circular sector in just 40 s [8,9]. However, further reducing this time by using a mechanical scan, could result too demanding both in terms of power consumption and system operation.

Installation and maintenance are other important aspects to consider in the overall assessment of a monitoring system. For example, even if the use of a large mechanical scanning system does not entail particular problems in easily accessible installations, or in well-equipped environments such as open-pit mines, it could turn out a strong limitation in remote regions operations.

Therefore, within the field of GB-InSAR systems, one of the most interesting lines of research is the replacement of mechanical scanning with some kind of Electronically Scanned Array (ESA). To this scope, Multiple Input Multiple Output (MIMO) radar are promising systems in the evolution of the GB-InSAR technology [10,11]. The MIMO principle of operation is to transmit and receive the radar signal alternately from various appropriately located elements. This strategy allows to synthesize an arbitrary antenna array, using a relatively small number of physical elements, thus limiting the complexity and cost of the whole system with respect to standard ESA [12].

Despite the innovations introduced by the MIMO strategy, the prototypes developed so far [10,11] still seem to suffer from major disadvantages in terms of production cost and ease of installation. To let GB-InSAR MIMO become a feasible and easily installable technology, IDS GeoRadar aimed to develop a system with a modular and integrated architecture. Modularity will help the installation procedure, allowing the sequential assembly of the various modules, rather than the whole system at the same time. Furthermore, the exploitation of highly integrated technologies such as microstrips and patch antennas, will lead to a cost reduction compared with other technologies such as coaxial cables and horn antennas [10].

In this paper, we present the SPARX array, a MIMO system developed by IDS GeoRadar, composed by 16 transmit and 16 receive antennas, organized in independent and integrated sub-modules and geometrically arranged in order to synthesize an equally spaced virtual array of 256 elements. 

## 2. MIMO Imaging System

A generic MIMO imaging system [13] is composed by $n_{TX}$ transmitting antennas placed in the positions $x_{m}$ and by $n_{RX}$ receiving antennas placed in the positions $y_{l}.$ Given a MIMO configuration consider a target located in r=re at a distance r far away respect to the system extent; the time of flight $\tau_{ml}$ that an electromagnetic signal takes to go from the *m*-th transmitter to the target and come back to the *l*-th receiver is approximately:

(1) $$\tau_{ml}≅\frac{2}{c}\;\left[r-\left(\frac{x_{m}+y_{l}}{2}\right)\cdot e\right];$$

that is equivalent to transmit and receive a signal from a unique antenna placed in the virtual phase center placed in $\left(x_{m}+y_{l}\right)/2$. Therefore, transmitting alternately from every transmitter and receiving alternately from every receiver, it can be generated a virtual array with $N=n_{TX}\cdot n_{RX}$ elements placed in the MIMO virtual phase centers. It can be noticed that, given a MIMO configuration, the dual one with transmitter and receiver swapped, generates the same phase centers positions. One of the main advantages of MIMO technique is that the number of virtual antennas grows as the square of the number of physical antennas; it is therefore possible to generate a large number of virtual elements, exploiting a relatively small number of physical antennas, with a significant cost and complexity reduction of the imaging system hardware.

### 2.1. MIMO Array Factor

Consider a MIMO array working with central wavelength λ, the corresponding array factor $F_{\mathrm{MIMO}}\left(e\right)$ is given by the product of the transmitting and the receiving array factors:

(2) $$F_{\mathrm{MIMO}}\left(e\right)=F_{TX}\left(e\right)F_{RX}\left(e\right)=\frac{1}{N}e^{i\frac{4\pi }{\lambda }r}\;\underset{m,l}{\sum }e^{i\frac{4\pi }{\lambda }\left(\frac{x_{m}+y_{l}}{2}\right)\cdot e}$$

**Figure 1:**
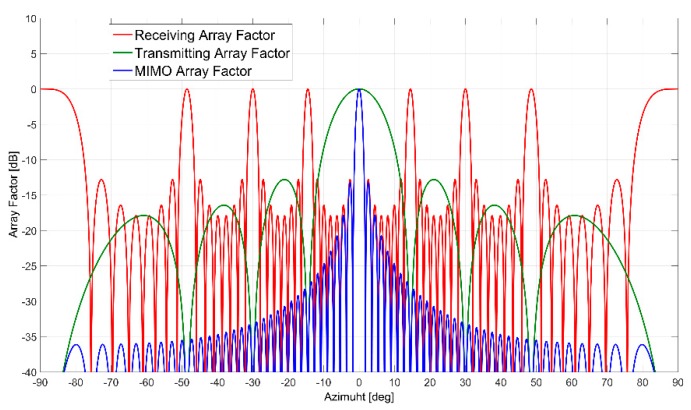
MIMO array factor (in blue), obtained from the product of a transmitting array factor (in green) and a receiving array factor (in red).

To avoid grating lobes in the MIMO array factor, it is necessary that $F_{TX}$ has a null in correspondence of every grating lobe of $F_{RX}$ and vice versa. In Figure 1 is shown an example of a typical MIMO array factor resulting from the product of a transmitting and receiving array factors, it can be noticed that all the grating lobes inside the receiving array factor are compensated by the nulls of the transmitting one.

In a physical array, different sources of errors cause deviations from the ideal model; this fact limits the array performances, typically degrading the SideLobe Level (*SLL*) of the point spread function. Assuming that, for a real system, the array factor F can be modeled as a random variable whose expectation value 〈F〉 is equal to the ideal one, then the *SLL* distribution can be read from the power of the statistical deviation δF=F−〈F〉:

(3) $$SLL=\langle {\left|\delta F\right|}^{2}\rangle .$$

In a MIMO array, the physical imperfections cause statistical deviations $\delta F_{TX}$, $\delta F_{RX}$ of the transmitting and the receiving array factors from the ideal ones; ignoring second order terms, the total deviation $\delta F_{\mathrm{MIMO}}$ from the ideal MIMO array factor can be expressed as:

(4) $$\delta F_{\mathrm{MIMO}}≃\delta F_{TX}\cdot F_{RX}+F_{TX}\cdot \delta F_{RX}.$$

Thus, in a MIMO array the *SLL* distribution is strongly dependent on the transmitting and the receiving array factors:

(5) $$SLL_{\mathrm{MIMO}}≃{\left|F_{RX}\right|}^{2}\langle {\left|\delta F_{TX}\right|}^{2}\rangle +{\left|F_{TX}\right|}^{2}\langle {\left|\delta F_{RX}\right|}^{2}\rangle +ℜ\left[\langle \delta F_{TX}\cdot \delta F_{RX}^{*}\rangle \delta F_{RX}\cdot \delta F_{TX}^{*}\right].$$

If the errors on the transmitter and the receiver are uncorrelated then the third term of this expansion vanishes. As a simple example, consider small and uncorrelated phase and amplitude errors on every transmitting and receiving element. From array theory it is well known that the effect of this kind of errors is to add to the sidelobes a uniform power level proportional to the mean square error:

(6) $$\langle {\left|\delta F_{TX}\right|}^{2}\rangle =\sigma_{TX}^{2}/n_{TX};\;\langle {\left|\delta F_{RX}\right|}^{2}\rangle =\sigma_{RX}^{2}/n_{RX}.$$

Using these relations to compute the MIMO SideLobe Level it is possible to gather that, in a MIMO array, small and uncorrelated phase and amplitude errors generate a non-uniform SideLobe Level:

(7) $$SLL_{\mathrm{MIMO}}≃{\left|F_{RX}\right|}^{2}\frac{\sigma_{TX}^{2}}{n_{TX}}+{\left|F_{TX}\right|}^{2}\frac{\sigma_{RX}^{2}}{n_{RX}}.$$

In particular, it can be noticed from the previous formula that, for a MIMO array, small errors produce strong sidelobes in the correspondence of transmitting and receiving grating lobes. To compensate this undesired effect, an efficient and reliable calibration procedure should be applied on the MIMO system. This reasoning can be easily generalized to other sources of errors like inaccuracy in the antennas placement, deformation of the system geometry, etc. The discussed behavior in the MIMO SideLobe Level has already been noticed in various experimental tests with MIMO arrays [14,15].

### 2.2. MIMO Uniform Linear Configurations

The simplest MIMO array configuration is composed by a uniformly spaced linear array of transmitting antennas parallel to a uniformly spaced linear array of receiving antennas [13,16]. Denoting with w the array axis direction, the antennas positions can be expressed as:

(8) $$x_{m}=m\cdot p_{TX}\cdot w+x_{0}\;m=1,\cdots ,n_{TX};\;y_{l}=l\cdot p_{RX}\cdot w+y_{0}\;l=1,\cdots ,n_{RX}.$$

where $p_{TX}$ and $p_{RX}$ are the transmitting and receiving spacing, respectively. The corresponding virtual phase centers are located in:

(9) $$\frac{m\cdot p_{TX}+l\cdot p_{RX}}{2}\cdot w+\frac{x_{0}+y_{0}}{2}.$$

**Figure 2:**
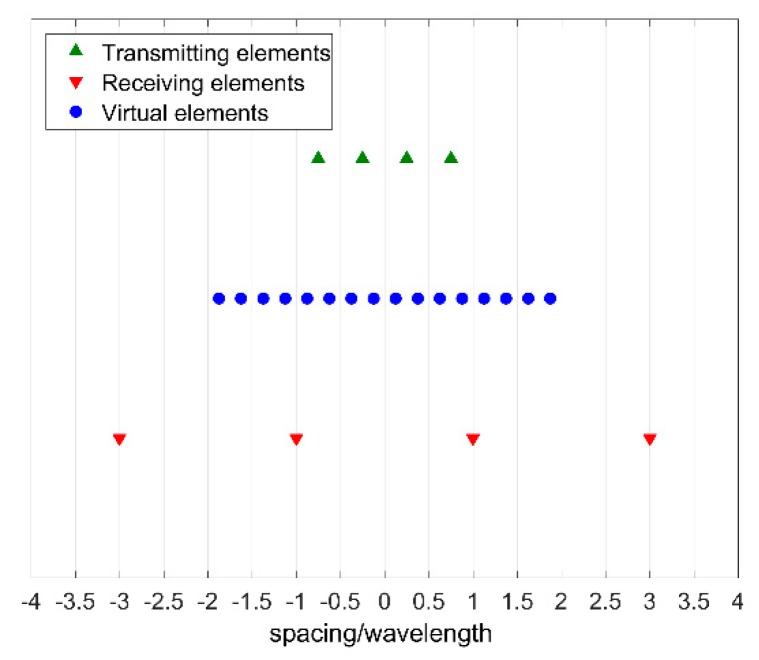
16 elements MIMO uniform linear array (in blue) obtained from 4 elements transmitting linear array and 4 elements receiving linear array.

Starting from this configuration, in order to generate a uniformly spaced array with $N=n_{TX}\cdot n_{RX}$ virtual elements it is necessary that the array spacing satisfy the relation $p_{RX}=n_{TX}\cdot p_{TX}$, or the dual one $p_{TX}=n_{RX}\cdot p_{RX}$_._ If one of these relations is satisfied, then the resulting linear array is uniformly spaced with a virtual spacing equal to $p_{TX}/2$, or $p_{RX}/2$ in the dual configuration (Figure 2).

In this case the smallest spacing between physical antennas is double the spacing of virtual elements, yet MIMO technique allows to create configuration with arbitrary large ratio between real spacing and the virtual spacing; to see this consider a MIMO configuration composed by a linear array of $n_{TX}=2k+1$ transmitting antennas uniformly spaced by $p_{TX}$, and a linear array of $n_{RX}\geq 2$ receiving antennas uniformly spaced by $n_{TX}\cdot p_{TX}/2$:

(10) $$x_{m}=m\cdot p_{TX}\cdot w+x_{0}\;m=1,\cdots ,2k+1;\;y_{l}=\left(2k+1\right)\cdot l\cdot \frac{p_{TX}}{2}\cdot w+y_{0}\;l=1,\cdots ,n_{RX}.$$

**Figure 3:**
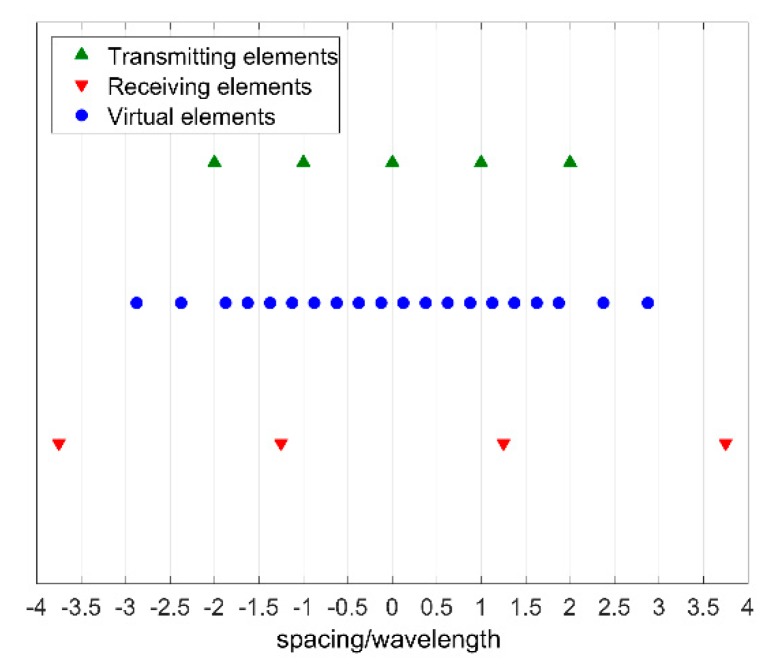
Twenty elements MIMO linear array (in blue) obtained from a five elements transmitting linear array and a four elements receiving linear array.

The corresponding virtual array is linear and contains a $N=n_{TX}\cdot \left(n_{RX}-1\right)+1$ elements sub-array uniformly spaced by $p_{TX}/4$. The uniformly spaced sub-array in Figure 3 is equivalent to the virtual array in Figure 2, however it has been obtained with five transmitting elements spaced by $p_{TX}$ instead of four transmitting elements spaced by $p_{TX}/2$; the second MIMO configuration although is require more physical antennas than the first one, allows to space apart the radiating elements decreasing mutual coupling effects.

## 3. SPARX Design

**Figure 4:**
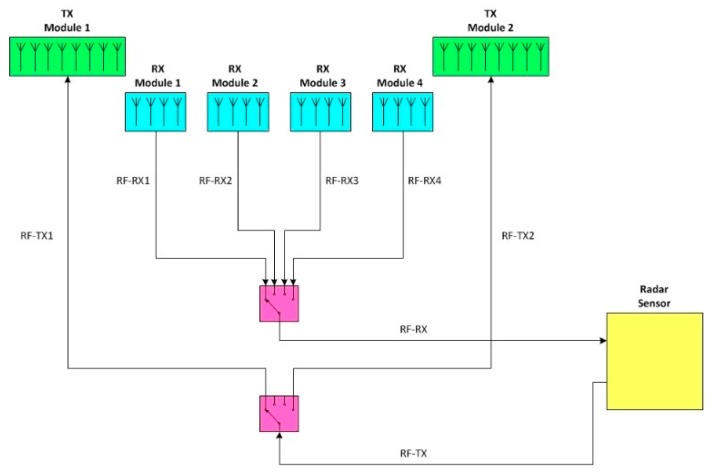
SPARX array block diagram.

Recently, in order to overcome the current limits of the ground based interferometric SAR systems, IDS GeoRadar developed SPARX: an X-band MIMO array. The SPARX array consists of 16 transmit and 16 receiver antennas, organized in independent sub-modules and geometrically arranged in order to synthesize a uniformly spaced virtual array of 256 elements. In Figure 4 is shown the SPARX block diagram: a radar sensor generates an X-band RF signal with a central frequency of 9.7 GHz and an instantaneous bandwidth of 275 MHz. The RF signal is transmitted to a Single Pole Double Throw (SPDT) switch stage and then switched between two transmitting antenna modules, each one consisted of an eight radiating elements switched array. The reflected signal is received by four receiving antenna modules each one composed by four radiating elements switched array and then collected by the Radar sensor through a Single Pole 4 Throw (SP4T) switch stage.

The two transmitting antenna arrays have a uniform spacing of 18 mm and the receiving antenna array has a uniform spacing of 144 mm; as discussed in the previous section, this configuration allow to generate 256 virtual elements uniformly spaced by 9 mm, that correspond to a uniform linear array with a λ/3.44 spacing.

**Figure 5:**
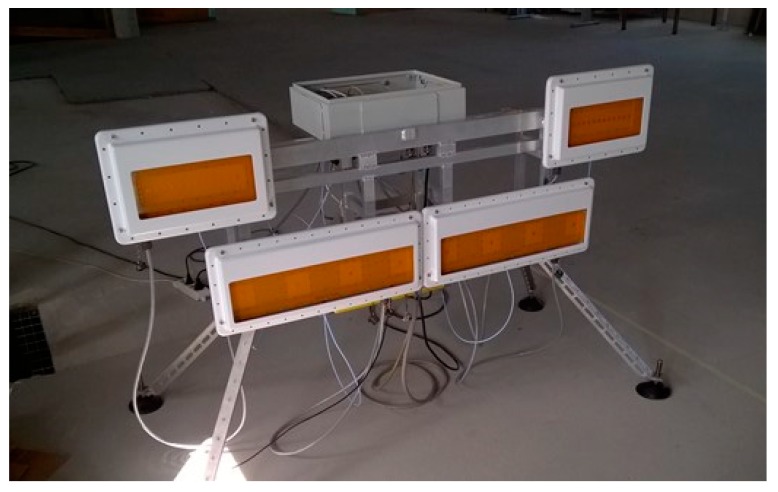
SPARX array prototype.

In Figure 5 it is shown a SPARX prototype with a reduced number of modules: two transmitting modules located in the upper external part and two receiving modules located in the inner lower part

### 3.1. Transmitting Antenna Module

**Figure 6:**
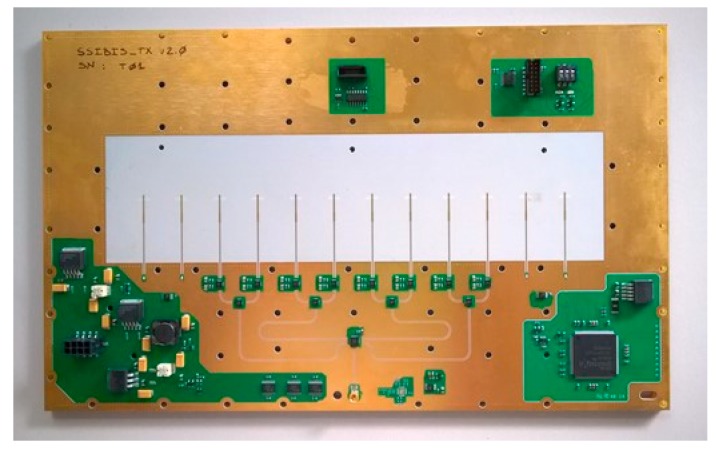
Transmitting antenna module.

**Figure 7:**
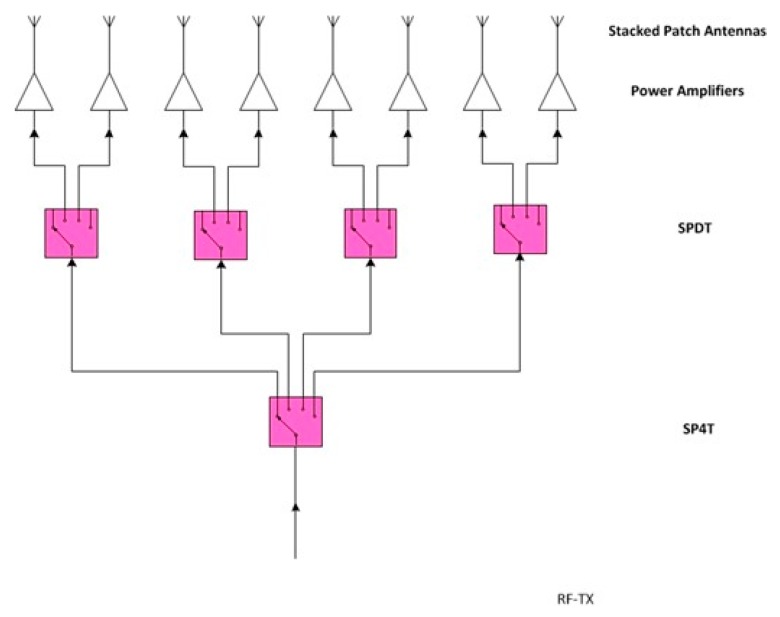
Transmitting antenna module block diagram.

The transmitting antenna module consists in a microstrip switch matrix that route the RF signal from a single input to eight stacked patch antennas. The microstrip transmission line and the patch antenna technology allow to fully integrate the module in a single PCB (Figure 6). In Figure 7 is shown the Transmitting Antenna Module block diagram: the incoming RF signal is transmitted by a microstrip and pass through a SP4T switch followed by four SPDT switches, this matrix allow to switch the signal between the eight antennas feed lines; immediately before every antenna a power amplifier compensate the losses of the microstrip transmission line and the switch stages

The radiating elements are stacked microstrip patch antennas with vertical polarization, properly designed by IDS’ laboratories in order to have 1 GHz bandwidth with a VSWR < 1.5 and a 3 dB beamwidth of 80° in the azimuth plane and 60° in the elevation plane. The total gain of the module including the power amplifier gain and transmission line losses is 15.7 dB.

### 3.2. Receiving Antenna Module

**Figure 8:**
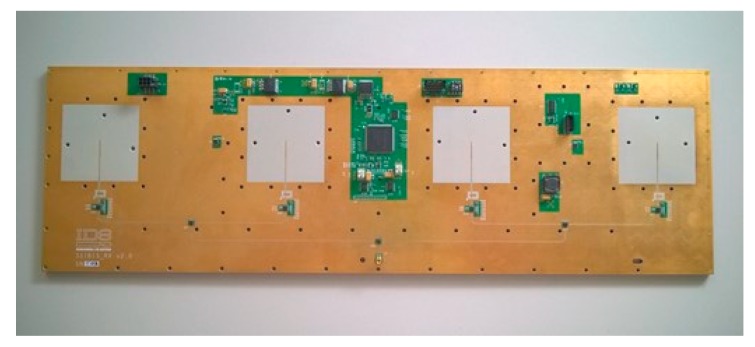
Receiving antenna module.

**Figure 9:**
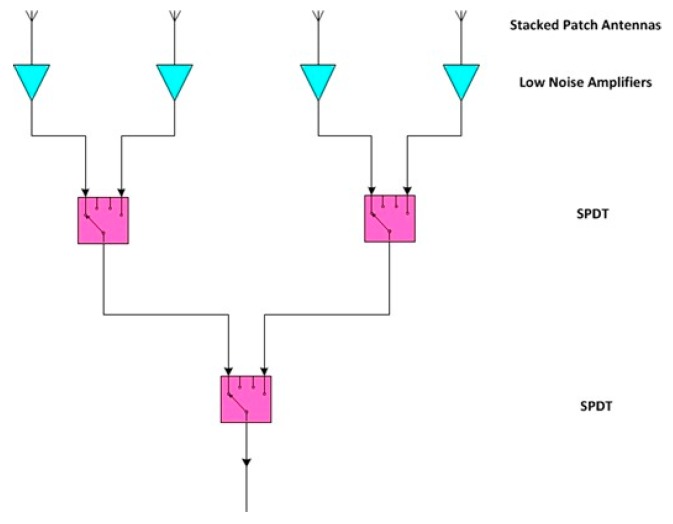
Receiving antenna module block diagram.

The receiving antenna module consists in a microstrip switch matrix that route the RF signal from four stacked patch antennas to a single output. The miscrostrip transmission line and the patch antenna technology allow to fully integrate the module in a single PCB (Figure 8). In Figure 9 is shown the receiving antenna module block diagram: immediately after every antenna a Low Noise Amplifier stage allow to keep low the noise figure of the system; the received RF signal pass through two SPDT switches followed by another SPDT switch, this matrix allow to switch the signal between the four antenna feed lines.

The radiating elements are stacked microstrip patch antennas with the same design of the transmitting one. The total gain of the module including the LNA gain and transmission line losses is 13.6 dB.

## 4. Field Test

The SPARX array operating principle has been tested with a reduced MIMO configuration, composed of two transmitting module and just one receiving module, for a total of 64 virtual channels. The system has been deployed in an external environment where it was possible to recognize various reflecting targets at different ranges and azimuth angles. The purpose of this preliminary test was to achieve the correct MIMO imaging in order to detect and identify all the relevant targets.

**Figure 10:**
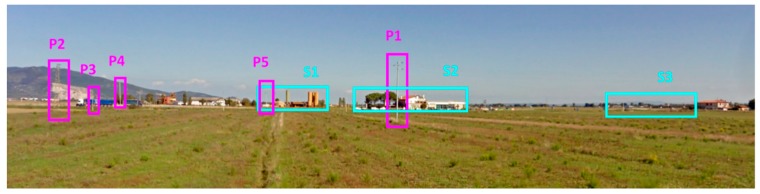
Acquisition scenario, SPARX point of view.

**Figure 11:**
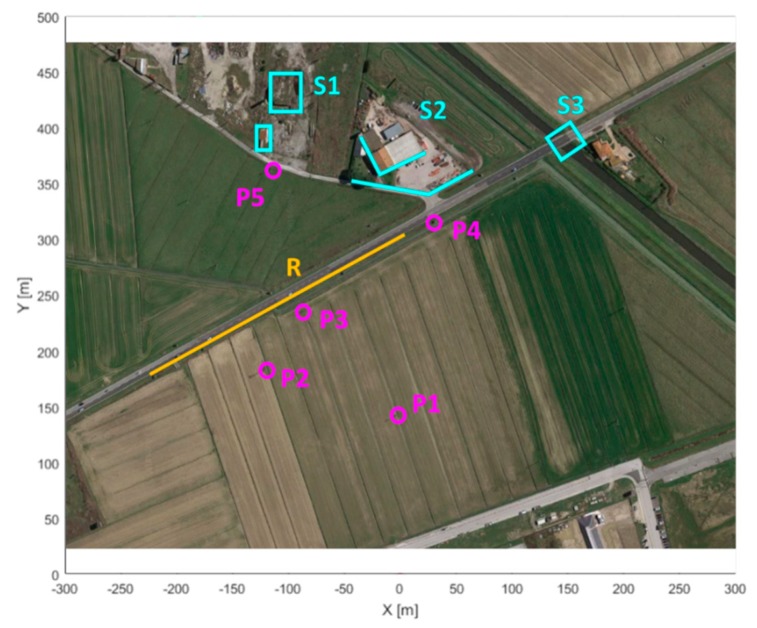
Acquisition scenario, top view.

The acquisition scenario from the SPARX point of view is shown in Figure 10, while in Figure 11 the same scenario from the top view is shown. In both images the reflecting targets were highlighted in various colors to better distinguish them; in particular it is possible to recognize a paved road R (in yellow), various metallic poles P1, P2, P3, P4 and P5 (in purple) and some structures S1, S2 and S3 (in light blue). In range, all the relevant targets are located between 140 m (P1) and 450 m (S1), while in azimuth they are included between −35° (P2) and +20° (S3).

## 5. Results

**Figure 12:**
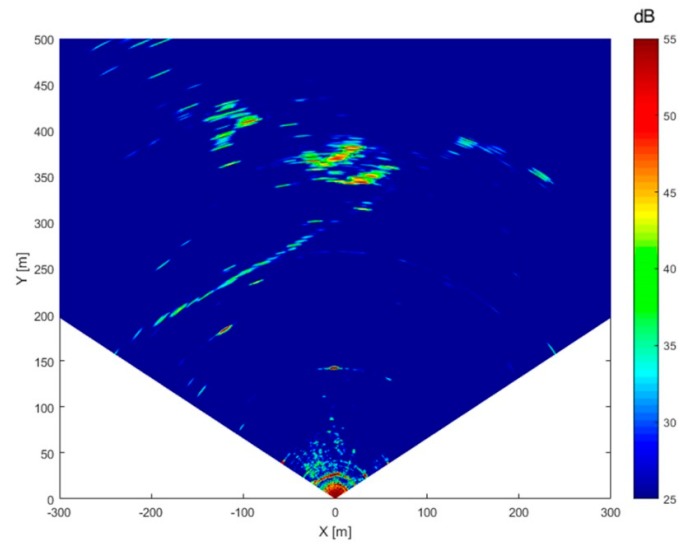
SPARX SNR estimated map (in dB).

After a standard range-azimuth data focusing, it was possible to extract the scenario power maps; in Figure 12 the resulting SNR map obtained from the SPARX acquisitions is shown. In this map target SNR levels are estimated comparing their powers with respect to the background thermal noise level. From this map it is possible to notice that all the relevant targets have been detected with a SNR greater than 35 dB, allowing an interferometric displacement measurement with precision greater than one tenth of a millimeter [5]. 

**Figure 13:**
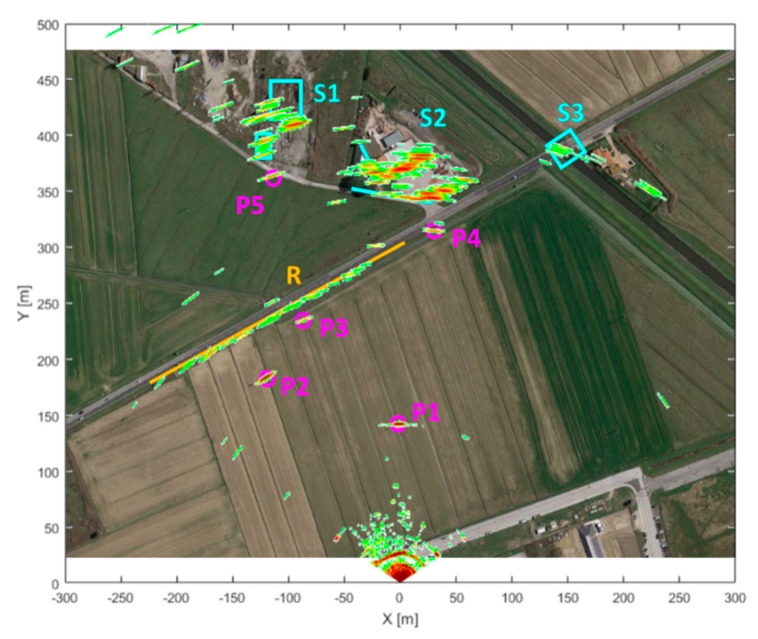
SPARX SNR estimated map superimposed on the acquisition scenario top view.

Overlapping the acquired SNR map (Figure 12) with the scenario top view (Figure 11) it is possible to identify every strong measured signal with a specific reflecting target inside the acquisition scenario (Figure 13); although it should be noted that, due to prototype’s low azimuth resolution, imaging of the farthest structures becomes rather coarse.

## 6. Conclusions

In this paper SPARX system have been introduced and described. It is a new MIMO system developed by IDS GeoRadar, in order to overcome some limitations of the current GB-InSAR systems. In particular, by decomposing the system into independent modules and using integrated technologies such as patch antennas and microstrip transmission line, SPARX development aims to reduce production costs and facilitate installation procedures compared to the current GB-InSAR MIMO prototypes. The field test conducted with the SPARX prototype showed that the MIMO imaging works effectively in detecting and identifying various target distributed inside the scenario.

However, it should be remarked that to obtain an azimuth resolution comparable to standard mechanical systems, a large number of modules is needed, thus greatly increasing the cost and complexity of the system. A possible solution to this difficulty is to exploit shorter wavelengths, in order to obtain high azimuth resolution compact systems [15], on the other hand, by reducing the transmitted wavelength, the operating range also decreases accordingly. From these considerations, it emerges that to reach a manufacturable GB-InSAR MIMO, the future developments will require a careful trade-off analysis between complexity and performances of the available technologies.
